# Construction and evaluation of leukemia suicide risk predictive model based on SEER database

**DOI:** 10.3389/fpsyt.2025.1506550

**Published:** 2025-02-21

**Authors:** Shan Zheng, Yuxin Tong, Jiayi Chen, Linlin Yang, Yamin Tan

**Affiliations:** ^1^ Department of Hematology, Zhejiang Cancer Hospital, Hangzhou, Zhejiang, China; ^2^ Hangzhou Institute of Medicine (HlM), Chinese Academy of Sciences, Hangzhou, China; ^3^ Postgraduate Training Base Alliance of Wenzhou Medical University (Zhejiang Cancer Hospital), Hangzhou, Zhejiang, China

**Keywords:** suicide, leukemia, predictive model, nomogram, hematolgy

## Abstract

**Background:**

A marked increase in suicide rate has been detected among individuals diagnosed with leukemia. Our research aimed to develop a predictive model intended for assessing the suicide risk in leukemia patients. This novel tool aims to optimize the process of pinpointing individuals at high risk within clinical environments, thereby guaranteeing the timely provision of targeted intervention strategies.

**Methods:**

Between 2000 and 2020, our study involved a cohort of 194584 leukemia patients, extracted from the Surveillance, Epidemiology, and End Results (SEER) database. These patients were randomly stratified into distinct training and validation cohorts. We utilized the Cox proportional hazards model to screen for influential variables and construct a predictive nomogram within the training set. The concordance index (C-index) and receiver operating characteristic (ROC) curves were employed to evaluate model’s discrimination, and calibration curves was used to assess the calibration ability. Furthermore, the validation set was utilized to conduct an internal validation process to ensure the robustness of nomogram.

**Results:**

Age, gender, race, residence, marital status, and histologic type were selected to construct the nomogram for predicting suicide risk of leukemia patients. In the training and validation sets, the C-indexes were 0.798 and 0.776, respectively. The calibration plots demonstrated a significant agreement between the predicted and actual outcomes. Ultimately, leukemia patients were divided into two groups, and Kaplan-Meier curves showed significant differences in the high- and low-risk groups, as confirmed in the validation set.

**Conclusions:**

We have successfully developed an intuitive and robust predictive model for assessing the suicide risk among leukemia patients. This model holds the potential to contribute to the reduction of preventable deaths.

## 1 Introduction

Suicide, defined as the deliberate act of ending one’s own life, represents a significant issue concern in global public health. In the United States, suicide ranked as the 11^th^ leading cause of death in 2021. The age-adjusted suicide rate witnessed an upward trajectory from 2001 (10.7 deaths per 100,000 standard population) to 2018 (14.2 deaths per 100,000 standard population). Subsequently, it declined for two consecutive years, reaching 13.5 deaths per 100,000 standard population in 2020, before rising again to 14.1 deaths per 100,000 standard population in 2021 (1). Cancer is the second leading cause of mortality in the United States, with an estimated 1,898,160 new cancer diagnoses and approximately 608,570 deaths attributed to cancer in the year 2021 (2). Although suicide accounts for merely 0.154% of deaths among cancer patients, the risk of suicide in this group was 4.4 times higher than that of the general population (3). Cancer can affect the immune system, potentially leading to depression and suicidal ideation, which in turn might trigger suicide (4). The risk factors for suicide in cancer patients encompass sociodemographic factors (age, gender, marital status and race), psychiatric disorders (pre-existing psychopathology, depression, anxiety and others), psychosocial factors (poor social support and loss of independence, feeling of being a burden), medical factors (advanced disease and poor prognosis and others), previous suicide attempt and suicidal thoughts (4). Studies have delved into the identification of pertinent risk factors for specific types of cancer and have consequently formulated predictive models tailored to these conditions, such as those for lymphoma (5), bladder cancer (6) and lung cancer (7).

Leukemia was the 15^th^ most commonly diagnosed cancer in 2018 and the 11^th^ leading cause of cancer-related deaths globally. There was a 25% increase in the total number of leukemia cases from 2005 to 2015 (8). Patients diagnosed with leukemia face an elevated risk of suicide for up to two years post-diagnosis. This heightened risk is attributed to the fact that many individuals transitioning to survivorship care endure long-term and late effects, both in physical and psychological, functional impairments, and poor quality of life (9). Identifying factors associated with the risk of suicide among leukemia patients is crucial. It enables early intervention by psychiatrists and has the potential to lower the rate of suicide. Although previous research have examined risk factors for suicide in leukemia patients (10, 11), no predictive model has yet been developed to quantify the individual suicide risk for this specific population.

In this study, we leveraged leukemia data from the SEER database to construct a predictive nomogram, which is designed to quantify the risk of suicide subsequent to a leukemia diagnosis among patients. We hope to assist clinicians in identifying those leukemia patients at high suicide risk during the process of diagnosis and treatment to reduce the unnecessary deaths of patients.

## 2 Materials and methods

### 2.1 Data resource

Patients diagnosed with leukemia were identified from the Surveillance, Epidemiology, and End Results (SEER) 17 Registries (version 8.4.3), which covers about 26.5% of the population in the United States. This extensive registry was programmed by the National Cancer Institute (NCI). International Classification of Diseases of Oncology (ICD-O)-3 codes 9800-9948, 9984 and 9987 were used to recruit patients diagnosed with leukemia. Patient variables incorporated a spectrum of demographic characteristics, including age (at diagnosis), gender, and race, alongside socio-demographic factors such as income, residence, and marital status (at diagnosis). Additionally, oncological characteristics were considered, comprising histologic type, chemotherapy, radiotherapy, and tumor sequence. The study included patients who met the following criteria: i) confirmation of diagnosis by microscopy at least, ii) complete survival follow-up data. Notably, patients with incomplete data on race, marital status, income, sequence number and residence were excluded. Finally, the study included a total of 194,584 patients with leukemia who fulfilled the inclusion criteria. In line with the 2016 World Health Organization (WHO) classification of myeloid tumors and acute leukemia, leukemias were classified into five categories: lymphocytic leukemia, acute myeloid leukemia, myeloproliferative neoplasms (MPN), myelodysplastic/myeloproliferative neoplasms (MDS/MPN), and not otherwise specific (NOS) (12). In the sequence number column, “one primary only” and “1st of 2 or more primaries” were defined as indicators of primary disease, while all other entries were categorized as non-primary disease. Regarding marital status at the time of diagnosis, leukemia patients were classified into three groups: married, single, and other, which included categories of unmarried or domestic partner, separated, widowed, and divorced. As for race, it was divided into four groups: White, Black, Hispanic and Other (including Non-Hispanic Asian or Pacific Islander and Non-Hispanic American Indian/Alaska Native). **Supplementary Figure S1** illustrates the flowchart of the screening process. The endpoint of the study was the cause of death from suicide, identified form the “COD to site recode” column for “suicide and self-inflicted injury”. The SEER database is a publicly accessible resource that safeguards patient privacy by employing unique patient IDs, thereby exempting the need for informed consent.

### 2.2 Statistical analysis

All data analyses were conducted using R Studio, version 4.4.0. The pooled dataset was randomly partitioned into a training set (N=136,208) and a validation set (N=58,376) at a ratio of 7:3. In the training set, we identified factors associated with suicide risk among leukemia patients to formulate a prediction model, which was subsequently evaluated it in the validation set. Student’s t-tests were utilized to analyze continuous characteristics, while the chi-square test or Fisher’s exact test was performed to compared categorical variables, as appropriate. Univariate Cox proportional hazards models were initially applied to identify potential risk factors, followed by multivariate Cox regression using the stepwise backward regression method. Variables with a P-value less than 0.1 in the univariate analysis were chosen for inclusion in the multivariate models. Within these multivariate models, a P-value of less than 0.05 was considered to denote statistical significance for the independent risk factors. The inclusion of covariates in the nomogram adhered to Harrell’s recommendation that the number of events should be at least ten times greater than the number of covariates (13). The risk scores for each predictor were derived from the multivariate Cox proportional hazards model and were graphically represented in a nomogram. This nomogram assigned a score to each predictive variable. The patient’s total risk score was calculated by summing these individual scores across all variables (7). The model’s discriminatory ability was evaluated through Harrell’s concordance index, known as the C-index, and the time-dependent receiver operating characteristic (ROC) curves. Both of the C-index and area under the curve (AUC) range from 0.5 to 1.0, where a score of 0.5 suggests that the model is equivalent to random guessing, and a value of 1.0 indicates perfect discrimination (14). A score above 0.7 shows that the model provides a reasonable estimation (15). The calibration was evaluated by calibration plots, with the Bootstrap method used for resample 1000 times. The closer the calibration curve is to the diagonal, the greater agreement between the observed and predicted probabilities. Subsequently, the training set was divided into two risk group: low-risk and high-risk—based on the median score derived from nomogram. Kaplan-Meier (K-M) curves were then used to assess and validate the differences in risk between two groups, within the training set and the validation set.

## 3 Results

### 3.1 Baseline characteristics

In our study, we recruited a total of 194584 leukemia patients who met the inclusion and exclusion criteria. Among the study population, 197 individuals, accounting for 1.01% of the total, were recorded as having died by suicide. **Table 1** displays the demographic, socio-demographic and oncological baseline characteristics of both the training and validation groups. No significant differences between training group and validation group (P > 0.05). Across the entire study population, the majority were males (58.2%), aged between 61 and 80 (43.8%), patients of white race (71.4%) and were married (53.9%). Moreover, more than a half of the patients (55.6%) accepted chemotherapy while a small proportion (3.9%) received radiotherapy. **Supplementary Table S1** delineates the characteristics of leukemia patients categorized by survival status, including those who were alive, those who died from causes other than suicide, and those who died by suicide. Statistically significant differences were observed among the three groups in terms of age, gender, race, marital status, radiotherapy, chemotherapy, sequence residence and income between the three groups.

**Table 1:** Baseline characteristics of patients in training and validation cohorts.

Variables	Training (%)	Validation (%)	Overall (%)	P value
	(N=136208)	(N=58376)	(N=194584)	
Age (years)				0.410
≤18	14026 (10.3)	6148 (10.5)	20174 (10.4)	
18-40	11626 (8.5)	5029 (8.6)	16655 (8.6)	
41-60	29958 (22.0)	12762 (21.9)	42720 (22.0)	
61-80	59766 (43.9)	25441 (43.6)	85207 (43.8)	
>80	20832 (15.3)	8996 (15.4)	29828 (15.3)	
Gender				0.607
Female	56937 (41.8)	24476 (41.9)	81413 (41.8)	
Male	79271 (58.2)	33900 (58.1)	113171 (58.2)	
Race				0.604
Black	10986 (8.1)	4755 (8.1)	15741 (8.1)	
White	97139 (71.3)	41711 (71.5)	133850 (71.4)	
Hispanic	19304 (14.2)	8144 (14.0)	27448 (14.1)	
Other^1^	8779 (6.4)	3766 (6.5)	12545 (6.4)	
Marital status				0.203
Married	73493 (54.0)	31311 (53.6)	104804 (53.9)	
Single	33937 (24.9)	14766 (25.3)	48703 (25.0)	
Other	28778 (21.1)	12299 (21.1)	41077 (21.1)	
Radiotherapy				0.938
No/Unknown/ Refused	130951 (96.1)	56128 (96.1)	187079 (96.1)	
Yes	5257 (3.9)	2248 (3.9)	7505 (3.9)	
Chemotherapy				0.243
No/Unknown	60566 (44.5)	25789 (44.2)	86355 (44.4)	
Yes	75642 (55.5)	32587 (55.8)	108229 (55.6)	
Sequence				0.614
Primary	106903 (78.5)	45877 (78.6)	152780 (78.5)	
Not primary	29305 (21.5)	12499 (21.4)	41804 (21.5)	
Histologic type				0.787
Lymphocytic leukemia	71205 (52.3)	30570 (52.4)	101775 (52.3)	
AML^2^	45169 (33.2)	19247 (33.0)	64416 (33.1)	
Leukemia-NOS	3232 (2.4)	1424 (2.4)	4656 (2.4)	
MDS/MPN	4482 (3.3)	1955 (3.3)	6437 (3.3)	
MPN	12120 (8.9)	5180 (8.9)	17300 (8.9)	
Residence				0.103
Large city^3^	80419 (59.0)	34568 (59.1)	114987 (59.1)	
Medium city	28383 (20.8)	12229 (20.9)	40612 (20.9)	
Small city	11000 (8.1)	4598 (7.9)	15598 (8.0)	
Suburbs	9580 (7.0)	4186 (7.2)	13766 (7.1)	
Rural	6826 (5.0)	2795 (4.8)	9621 (4.9)	
Income				0.896
>$70000	72311 (53.1)	31044 (53.2)	103355 (53.1)	
$55000-$70000	43418 (31.9)	18599 (31.9)	62017 (31.9)	
<$55000	20479 (15.0)	8733 (15.0)	29212 (15.0)	

^1^including Non-Hispanic Asian or Pacific Islander and Non-Hispanic American Indian/Alaska Native.

^2^AML: acute myeloid leukemia, MDS/MPN: myelodysplastic/myeloproliferative neoplasms, Leukemia-NOS: including leukemia-not otherwise specified (NOS) and myeloid leukemia-NOS.

^3^Large city: counties in metropolitan areas ge 1 million pop, medium city: counties in metropolitan areas of 250,000 to 1 million pop, small city: counties in metropolitan areas of lt 250 thousand pop, suburbs: nonmetropolitan counties adjacent to a metropolitan area, rural: nonmetropolitan counties not adjacent to a metropolitan area.

### 3.2 Variables selection

In the univariate Cox regression analysis, a total of nine variables including age (at diagnosis), gender, race, residence, marital status (at diagnosis), histologic type, chemotherapy, sequence and income demonstrated significant associations with the risk of suicide. Advancing to the multivariate Cox regression analysis, we identified age (at diagnosis), gender, race, residence, marital status (at diagnosis) and histologic type as independent prognostic factors predictive of suicide incidence among leukemia patients, as detailed in **Table 2**.

**Table 2:** Univariate and multivariate Cox proportional hazards regression analysis.

Variables	Univariate Cox	P-value	Multivariate Cox	P-value
	HR (95%CI)		HR (95%CI)	
Age				
<18	Reference			
18-40	3.35 (1.01-11.14)	**0.048** ^4^	2.98 (0.86-10.24)	0.084
40-60	7.36 (2.64-20.50)	**<0.001**	6.35 (2.07-19.49)	**0.001**
60-80	7.02 (2.54-19.48)	**<0.001**	6.03 (1.94-18.77)	**0.002**
>80	12.23 (4.13-36.25)	**<0.001**	10.58 (3.14-35.58)	**<0.001**
Gender				
Female	Reference			
Male	5.74 (3.36-9.83)	**<0.001**	7.05 (4.08-12.18)	**<0.001**
Race				
Black	Reference			
White	3.20 (1.18-8.66)	**0.022**	2.84 (1.04-7.74)	**0.041**
Hispanic	1.13 (0.35-3.66)	0.842	1.75 (0.54-5.71)	0.356
Other^1^	0.31 (0.03-2.78)	0.296	0.38 (0.04-3.44)	0.391
Marital status				
Married	Reference			
Single	0.52 (0.33-0.83)	**0.006**	1.44 (0.84-2.44)	0.182
Other	1.67 (1.13 -2.48)	**0.011**	2.41 (1.60-3.63)	**<0.001**
Radiotherapy				
No/Unknown/ Refused	Reference			
Yes	1.10 (0.51-2.36)	0.804	–	–
Chemotherapy				
No/Unknown	Reference			
Yes	0.66 (0.47-0.92)	**0.016**	–	**-**
Sequence				
Primary	Reference			
Not primary	1.60 (1.05-2.44)	**0.029**	–	–
Histologic Type				
Lymphocytic leukemia	Reference			
AML^2^	1.46 (0.97-2.20)	**0.071**	1.93 (1.26-2.95)	**0.002**
Leukemia-NOS	0.60 (0.08-4.32)	0.613	0.67 (0.09-4.85)	0.694
MDS/MPN	1.32 (0.41-4.19)	0.641	1.05 (0.33-3.34)	0.938
MPN	1.18 (0.70-2.00)	0.529	1.29 (0.76-2.21)	0.350
Income				
>70000	Reference			
55000-70000	1.15 (0.78-1.69)	0.491	–	–
<55000	1.83 (1.18-2.83)	**0.007**	–	–
Residence				
Large city^3^	Reference			
Medium city	1.38 (0.88-2.16)	0.160	1.34 (0.85-2.10)	0.203
Small city	2.65 (1.61-4.35)	**<0.001**	2.46 (1.49-4.05)	**<0.001**
Suburbs	1.96 (1.07-3.57)	**0.029**	1.69 (0.93-3.10)	0.087
Rural	2.75 (1.51-5.02)	**<0.001**	2.41 (1.32-4.40)	**0.004**

^1^Other including Non-Hispanic Asian or Pacific Islander and Non-Hispanic American Indian/Alaska Native.

^2^AML: acute myeloid leukemia, MDS/MPN: myelodysplastic/myeloproliferative neoplasms, Leukemia-NOS: including leukemia-not otherwise specified (NOS) and myeloid leukemia-NOS.

^3^Large city: counties in metropolitan areas ge 1 million pop, medium city: counties in metropolitan areas of 250,000 to 1 million pop, small city: counties in metropolitan areas of lt 250 thousand pop, suburbs: nonmetropolitan counties adjacent to a metropolitan area, rural: nonmetropolitan counties not adjacent to a metropolitan area.

^4^Bold values are considered statistically significant.

### 3.3 Model construction and validation

**Figure 1:**
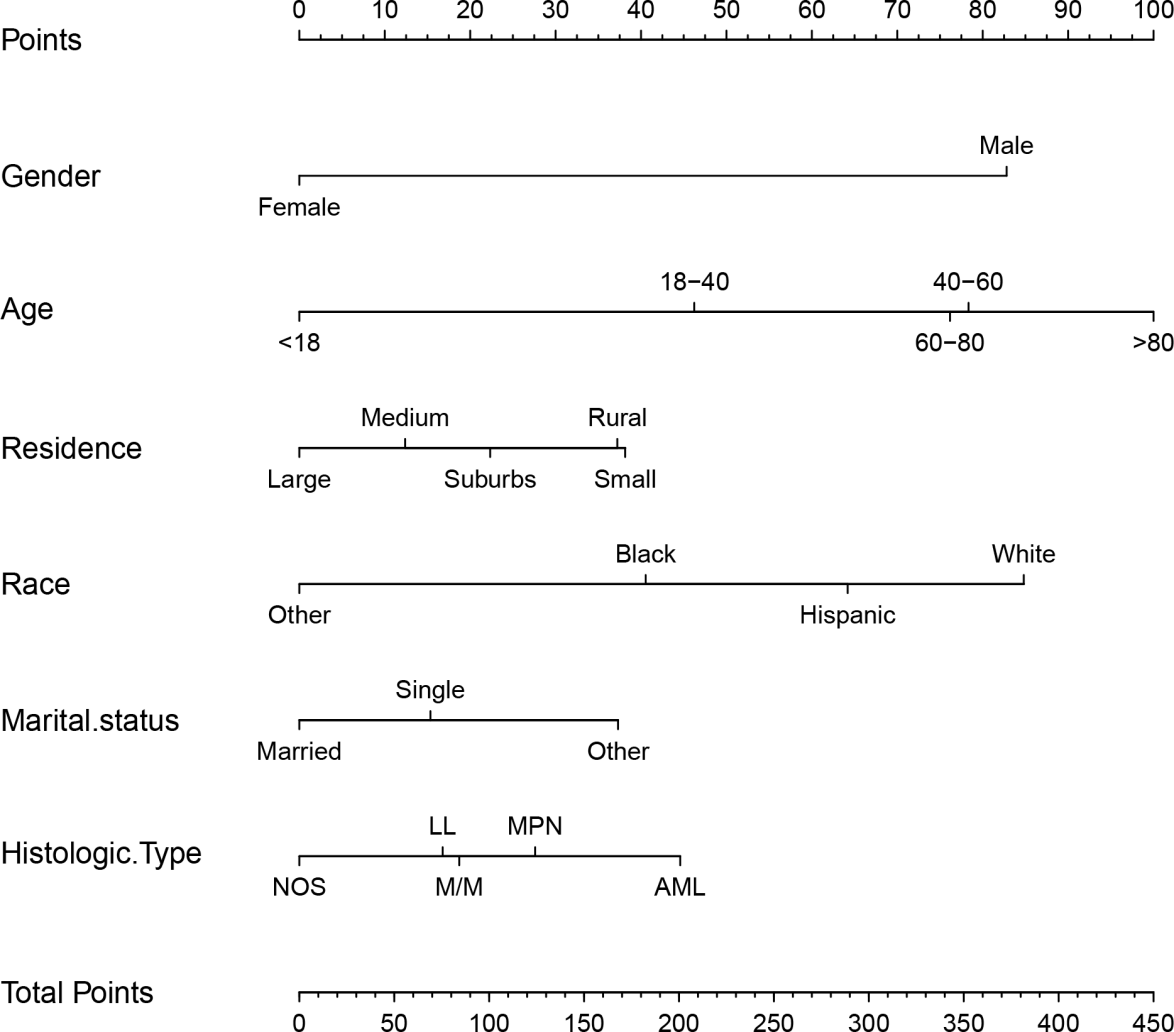
The nomogram calculating the total risk score for suicide risk in leukemia patients. M/M indicates MDS/MPN, NOS including leukemia-NOS and mixed phenotypic leukemia. Large city: counties in metropolitan areas ge 1 million pop, medium city: counties in metropolitan areas of 250,000 to 1 million pop, small city: counties in metropolitan areas of lt 250 thousand pop, suburbs: nonmetropolitan counties adjacent to a metropolitan area, rural: nonmetropolitan counties not adjacent to a metropolitan area.

**Figure 2:**
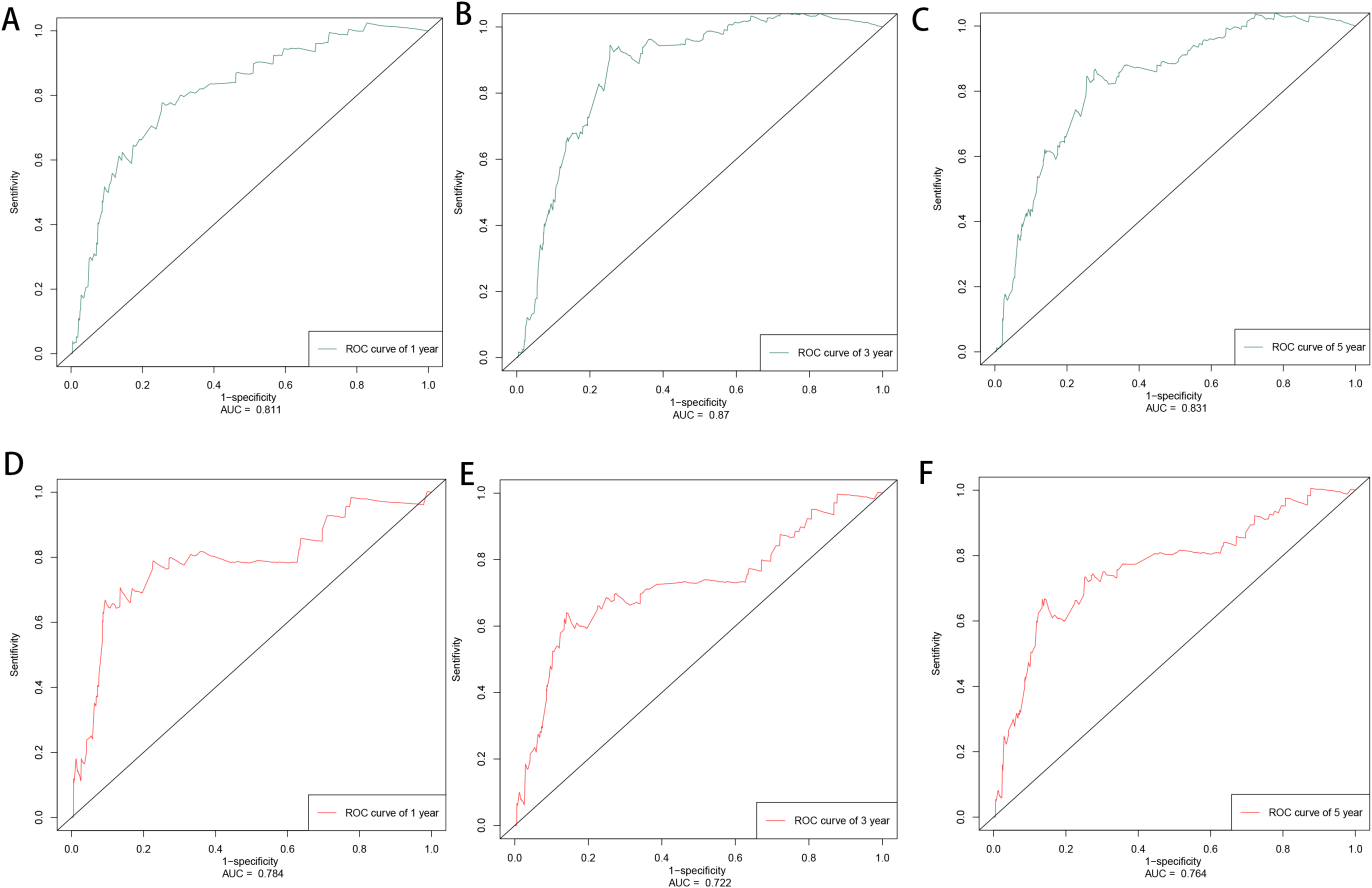
The time-dependent ROC curves of training set in 1, 3, 5 years **(A–C)** and validation set in 1, 3, 5 years **(D–F)**.

**Figure 3:**
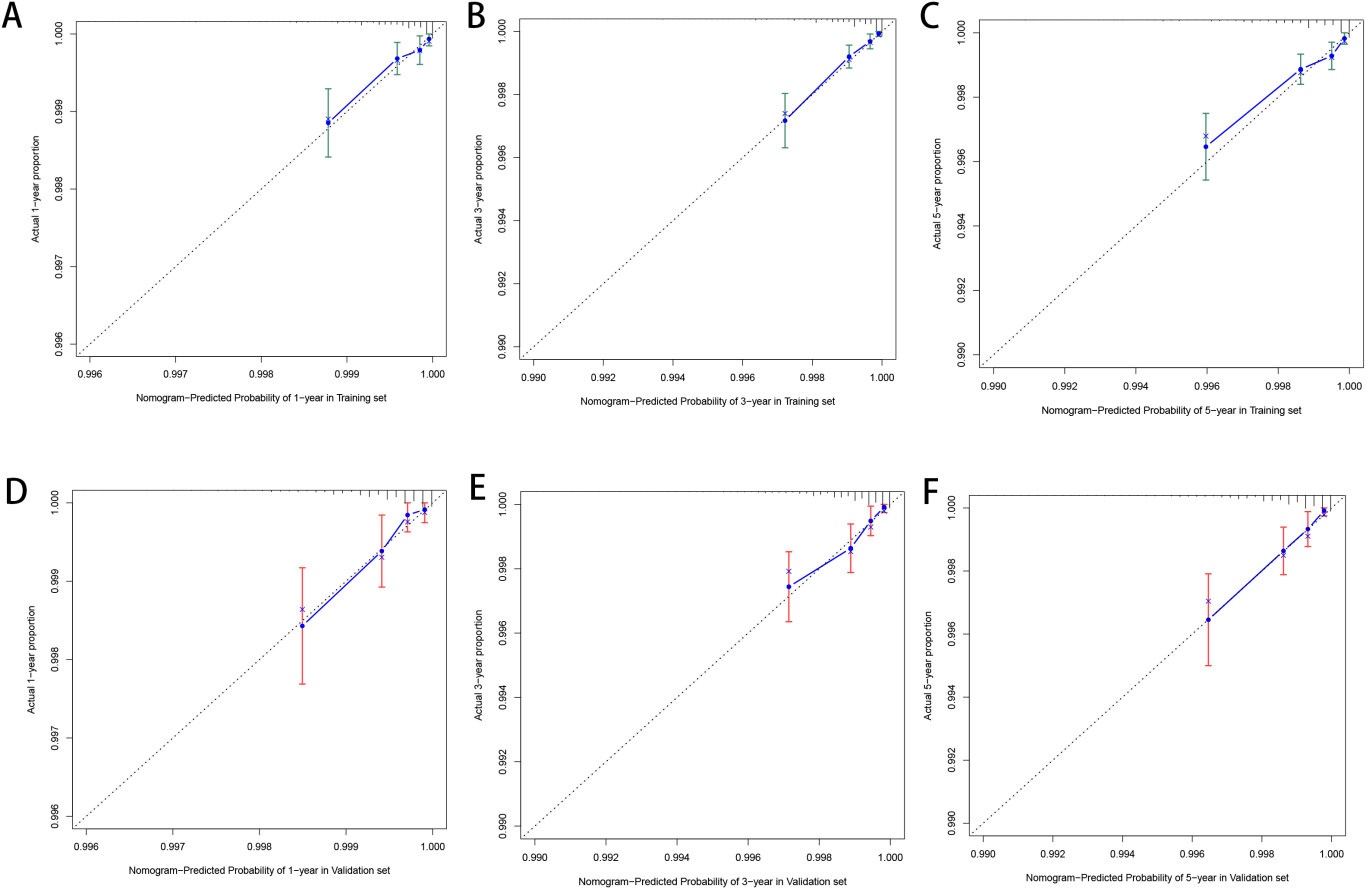
The calibration plot for prediction in the training **(A–C)** and validation cohorts **(D–F)**.

**Figure 4:**
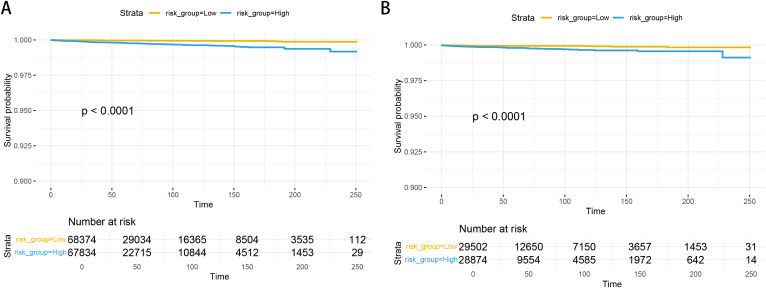
The K-M curves in the training set **(A)** and validation set **(B)**.

Based on the factors derived from the multi-factor Cox regression model, we constructed a predictive nomogram, as depicted in **Figure 1**. The C-index was 0.798 (95% confidence interval [CI]: 0.759-0.837) for the training set, while for the validation set, it was 0.776 (95% CI: 0.715-0.837). The AUCs for the 1-, 3- and 5- year were 0.811, 0.87 and 0.831 in training set, respectively and in the validation set, these values were 0.784, 0.722 and 0.764 at the corresponding time points, demonstrating the nomogram’s great discrimination (**Figure 2**). Calibration curves for the nomogram revealed a favorable degree of consistency between the predicted and observed probabilities in both the training and validation cohorts (**Figure 3**). Finally, we processed a risk stratification based on the total points calculated using the nomogram. Leukemia patients were therefore divided into two distinct risk groups, low-risk group and high-risk group, based on the median number of 245. The Kaplan‐Meier (K-M) curves exhibited a significant difference in suicide survival rates between the two groups, which was validated in the validation set (**Figure 4**).

## 4 Discussion

A cancer diagnosis often leads patients to endure significant distress and exhibit psychiatric symptoms, thereby indicating an elevated risk of suicide (16). Leukemia, a prevalent malignancy affecting the hematologic system, exhibits a suicide rate that surpasses that of the general population, with a standardized mortality ratio (SMR) of 2.16 (11). In our study, based on the SEER database, through univariate Cox regression and multivariate Cox regression, we finally screened six factors including age, gender, race, residence, marital status and histologic type, affecting suicide among leukemia patients. Subsequently, we constructed a Nomogram. The C-index and time-dependent ROC curves demonstrated outstanding discrimination capabilities, while the calibration curves confirmed a high degree of agreement between the predicted and observed survival probabilities. Diverging from previous studies that focused solely on risk factors identification, our predictive model assigns a quantifiable suicide risk score for each patient. To the best of our knowledge, this was the first predictive model specifically designed to quantify suicide risk in leukemia patients. This innovative approach allows hematologists to more accurately identify individuals at increased risk for suicide during clinical consultations, allowing for early intervention.

Males are generally at a significantly higher risk of suicide compared to females. This assertion is supported by previous analyses of risk factors for suicide across various cancer types, as well as specific findings related to leukemia patients (10, 11, 17). During the period from 2001 to 2021, the suicide rate among males in the United States was reported to be three to four and a half times higher than that of females (1). Our findings align with this trend, revealing that the suicide risk among males is 7.05 times higher than females. This disparity may stem from the higher prevalence of aggression and alcoholism are more prevalent among males, both of which have been identified as strong precipitating factors for suicide, as supported by high-quality evidence (4, 18). Analysis of data from the National Violent Death Reporting System in the United States reveals that approximately one in five suicides is attributable to alcohol use. In 2021, the proportion of male suicides attributed to alcohol-associated factors (AAF) was significantly higher at 20.2% compared to 17.8% among females (P <.001) (19). Additionally, smokers demonstrated higher suicide-related risks than non-smokers (20), which may further contribute to the increased suicide risk observed in males. In the context of AML, males are more often categorized into the adverse-risk group according to European LeukemiaNet criteria. However, when comparing *SF3B1* mutations, which are male-specific adverse outcome prognosticators, to *SF3B1* mutations carried by females, males with these mutations have worse OS outcomes (21), This worse disease prognosis may suggest a higher suicide risk among these patients (5).

Among marital status categories, married patients have a lower risk of suicide compared to those who are single (HR: 1.44) and those belonging to other marital status groups (HR: 2.41), including unmarried or in domestic partnerships, separated, widowed, and divorced. Chen et al. discovered that suicide rates increased over time among individuals who were divorced/separated, never-married, or widowed, and that being married served as a protective factor against suicide risk (22). This finding was consistent with the results of our study. The influence of marital status on suicide rates was consistent and stable, and the reason of this relationship may be linked to the bonds of social support. Social support has been recognized as an environmental risk factor for suicide, and the lack of it among unmarried or divorced individuals may contribute to their increased suicide risk (18). Furthermore, for separated men, factors such as lower educational attainment, shame associated with separation, and stress from legal negotiations, particularly regarding property/financial issues, have been linked to the development of serious suicidal ideation (23).

White people had the highest risk of suicide with reports indicating that the suicide rate for white people was 13 per 100,000 person-years (24). In our study, being white people was identified as a risk factor when compared to Black, Hispanic, Asian or Pacific Islander and American Indian/Alaska Native papulations. Differences may be explained by variations in psychiatric disorders and lower risk factors prior to arriving in the United States, as many Hispanic and Asian individuals are immigrants (25). It is currently believed that Black people have higher levels of religious belief and family cohesion, which serve as protective factors against suicide risk. Furthermore, suicides among Black people are more likely to be misclassified as accidents or undetermined, potentially underestimating the suicide rate within this population (26).

Among patients diagnosed with leukemia, those with AML demonstrated the highest suicide risk, despite lymphocytic leukemia constituting the majority of the study population. AML is the most common type of leukemia in adults, particularly affecting older patients more severely than their younger counterparts, featured by rapid progression and poor prognosis. In our investigation, the proportion of individuals who died within 6 months was significantly higher in the AML group (47.2%) compared to lymphocytic leukemia group (10.3%). A poor prognosis was strongly correlated with suicide risk in the initial stages following a cancer diagnosis (27). Additionally, secondary leukemia, which accounted for 40% of all AML or myelodysplastic syndrome (MDS) cases (28), may also contribute to this heightened risk. Patients with a history of prior tumors or other medical conditions who have undergone treatment are understandably at a higher risk of suicide than those with primary leukemia. Moreover, for patients who had antecedent hematologic conditions tend to exhibited poorer outcome than those with *de novo* AML.

It is noteworthy that older adults, particularly men over the age of 80, are at a higher risk of suicide compared to other age groups (29), a trend corroborated by both our study and previous research (30). This increased susceptibility can be attributed to various factors, including the loss of loved ones, loneliness, and the accumulation of physical illnesses that often accompany advancing age (29). Mood disorders, especially major depression, are prevalent among older adults who die by suicide, with estimates ranging from 54% to 87% (31) In the context of cancer patients, the prevalence of depression was even more pronounced, being four times higher than that of the general population, and is associated with adverse outcomes such as reduced adherence to treatment and increased mortality within the oncology setting (31). Among elderly, a significant number are unemployed (32), may also contribute to the increased risk of suicide in this population. Of note, though the population under 18 years old exhibited the lowest suicide risk in our study, there is a concerning global trend of increasing suicide risk within this demographic. Consequently, it is imperative to prioritize the mental health of children diagnosed with leukemia.

The geographic residence of leukemia patients emerged as a significant factor influencing their risk of suicide in our study. This phenomenon may be attributed to the poor mental health functioning observed among cancer survivors residing in rural areas, who exhibit heightened symptoms of anxiety, and depression, greater distress and more emotional problems compared to their counterparts in non-rural settings (33). Furthermore, individuals in rural locations often face limited access to mental health resources. However, it is important to note that residency is not static, due to the limitations of the SEER database, this study was unable to assess the impact of population mobility and geographical changes on the suicide risk among leukemia patients.

Although the nomogram developed in this study demonstrated strong performance, we acknowledge several limitations. Firstly, the SEER database does not provide information regarding hematopoietic stem cell transplantation (HSCT) for leukemia patients, while the significant role HSCT plays in the treatment process of leukemia. The elevated suicide rates in patients undergoing autologous HSCT have been associated with a higher rate of relapse, whereas the increased suicide rates in those receiving allogeneic HSCT were more attributable to chronic graft-versus-host disease (34). Additionally, patients who have undergone HSCT may be at risk for major depression (35). Secondly, The SEER database does not encompass information on drug use, psychiatric comorbidities, environmental exposures, and occupational background—factors that are widely acknowledged for their contribution to suicide risk. Consequently, these elements could not be integrated into our nomogram. Lastly, although we have established and validated our model within a large cohort, we have not conducted external validation of the model. The SEER data only contains data for Americans, so external validation in other countries and populations is necessary to verify generalizability and credibility. Traditionally, doctors assess a patient’s risk of suicide through interviews, which is a subjective method. Our model offers a specific scoring system for leukemia patients to quantify their risk of suicide. Regular screening for suicide risk is essential, and those identified as being at high risk should be promptly referred to a psychiatrist for treatment. Additionally, communication with patients and their families is crucial to understand any changes in the patient’s physical and psychological state.

## 5 Conclusions

Suicide represents a significant global public health concern, resulting in numerous fatalities worldwide. Notably, its incidence among individuals with cancer is higher compared to the general populace. Utilizing the SEER database, we employed the Cox proportional hazards model to pinpoint six factors—namely, age, gender, race, residence, histological type, and marital status—that are linked to suicidality among leukemia patients. Consequently, we formulated a straightforward yet reliable model that aids specialists in promptly identifying individuals at heightened risk of suicide, thereby enabling them to enhance the patients’ prognosis.

## Data Availability

Publicly available datasets were analyzed in this study. This data can be found here: https://seer.cancer.gov/.

## Supplementary material

Supplementary Figure S1 The flow chart of screening data.
