# Supplementary figures and images for: Construction and evaluation of leukemia suicide risk predictive model based on SEER database
Source: Front Psychiatry. 2025 Feb 21;16:1506550. doi: 10.3389/fpsyt.2025.1506550 (PMC11885224; doi:10.3389/fpsyt.2025.1506550)

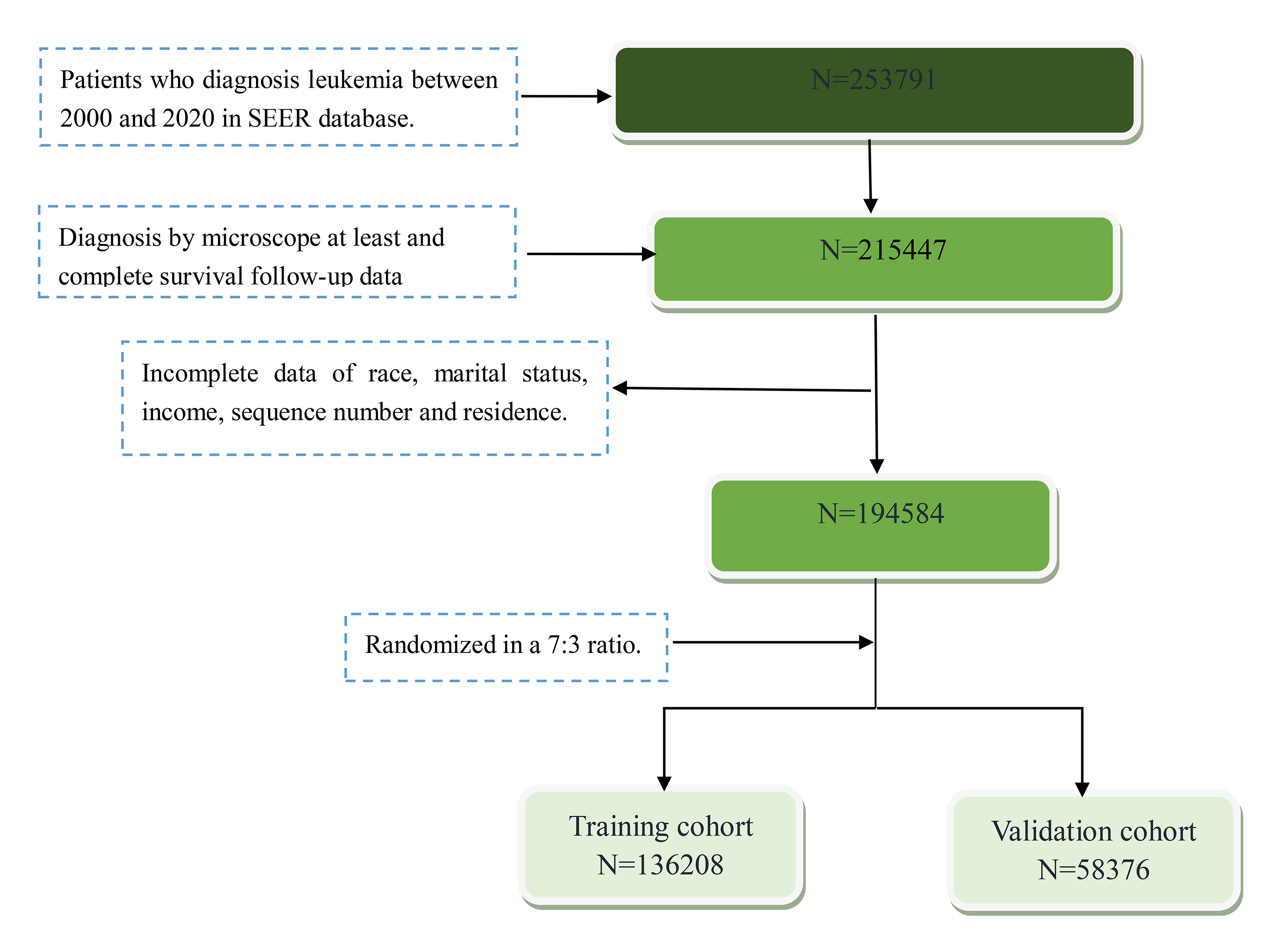

Supplement: Supplementary Figure S1 — The flow chart of screening data. [file Image1.tif]
