# Supplementary material for: Construction and evaluation of leukemia suicide risk predictive model based on SEER database
Source: Front Psychiatry. 2025 Feb 21;16:1506550. doi: 10.3389/fpsyt.2025.1506550 (PMC11885224; doi:10.3389/fpsyt.2025.1506550)
Supplement: Supplementary file 2 [file Table1.docx]

**Table S1.** **characteristics among alive, non-suicide death and suicide death leukemia patients.**

| **Variables** | **Alive (%)** | **Non-suicide (%)** | **Suicide (%)** | **P-value** |
| --- | --- | --- | --- | --- |
| **Age** |  |  |  | **<0.001** |
| <18 | 16799 (18.9) | 3369 (3.2) | 6 (3.0) |  |
| 18-40 | 110570 (11.9) | 6071 (5.8) | 14 (7.1) |  |
| 40-60 | 24878 (27.9) | 17775 (16.8) | 67 (34.0) |  |
| 60-80 | 32386 (36.4) | 52742 (50.1) | 79 (40.1) |  |
| >80 | 4411 (5.0) | 25386 (24.1) | 31 (15.7) |  |
| **Gender** |  |  |  | **<0.001** |
| Female | 38005 (42.7) | 43379 (41.2) | 29 (14.7) |  |
| Male | 51039 (57.3) | 61964 (58.8) | 168 (85.3) |  |
| **Race** |  |  |  | **<0.001** |
| Black | 6709 (7.5) | 9026 (8.6) | 6 (3.0) |  |
| White | 60989 (68.4) | 77774 (73.8) | 178 (90.4) |  |
| Hispanic | 15475 (17.4) | 11962 (11.4) | 11 (5.6) |  |
| Other^1^ | 5962 (6.7) | 6581 (6.2) | 2(1.0) |  |
| **Marital status** |  |  |  | **<0.001** |
| Married | 46315 (52.0) | 58380 (55.4) | 109 (55.3) |  |
| Single | 30672 (33.4) | 17991 (17.1) | 40 (20.3) |  |
| Other | 12057 (13.5) | 28972 (27.5) | 48 (24.4) |  |
| **Radiotherapy** |  |  |  | **<0.001** |
| No/Unknown/  Refused | 84934 (95.4) | 101956 (96.8) | 189 (95.9) |  |
| Yes | 4110 (4.6) | 3387 (3.2) | 8 (4.1) |  |
| **Chemotherapy** |  |  |  |  |
| No/Unknown | 36857 (41.4) | 49388 (46.9) | 110 (55.8) | **<0.001** |
| Yes | 52187 (58.6) | 55955 (53.1) | 87 (44.2) |  |
| **Sequence** |  |  |  | **<0.001** |
| Primary | 77122 (86.6) | 75506 (71.7) | 152 (77.2) |  |
| Not primary | 11922 (13.4) | 29837 (28.3) | 45 (22.8) |  |
| **Histologic Type** |  |  |  |  |
| Lymphocytic leukemia | 60374 (67.8) | 41287 (39.2) | 114 (57.9) |  |
| AML^2^ | 15205 (17.1) | 49163 (46.7) | 48 (24.4) |  |
| Leukemia-NOS | 1019 (1.1) | 3634 (3.4) | 3 (1.5) |  |
| MDS/MPN | 1345 (1.5) | 5086 (4.8) | 6 (3.0) |  |
| MPN | 4411 (5.0) | 25386 (24.1) | 31 (15.7) |  |
| **Income** |  |  |  | **<0.001** |
| >70000 | 49851 (56.0) | 53416 (50.7) | 88 (44.7) |  |
| 55000-70000 | 27233 (30.6) | 34721 (33.0) | 63 (32.0) |  |
| <55000 | 11960 (13.4) | 17206 (16.3) | 29212 (15.0) |  |
| **Residence** |  |  |  | **<0.001** |
| Large city^3^ | 53654 (60.3) | 61248 (58.1) | 85 (43.1) |  |
| Medium city | 18762 (21.1) | 21803 (20.7) | 47 (23.9) |  |
| Small city | 6819 (7.7) | 8751 (8.3) | 28 (14.2) |  |
| Suburbs | 5754 (6.5) | 7991 (7.6) | 21 (10.7) |  |
| Rural | 4055 (4.6) | 5550 (5.3) | 16 (8.1) |  |

^1^Other including Non-Hispanic Asian or Pacific Islander and Non-Hispanic American Indian/Alaska Native.

^2^AML: acute myeloid leukemia, MDS/MPN: myelodysplastic/myeloproliferative neoplasms, Leukemia-NOS: including leukemia-not otherwise specified (NOS) and myeloid leukemia-NOS.

^3^Large city: counties in metropolitan areas ge 1 million pop, medium city: counties in metropolitan areas of 250,000 to 1 million pop, small city: counties in metropolitan areas of lt 250 thousand pop, suburbs: nonmetropolitan counties adjacent to a metropolitan area, rural: nonmetropolitan counties not adjacent to a metropolitan area.
